# Supplementary material for: Cell type-specific transcriptional programs in mouse prefrontal cortex during adolescence and addiction
Source: Nat Commun. 2019 Sep 13;10:4169. doi: 10.1038/s41467-019-12054-3 (PMC6744514; doi:10.1038/s41467-019-12054-3)
Supplement: Supplementary file 1 — Supplementary Information [file 41467_2019_12054_MOESM1_ESM.pdf]

Supplementary Information

Cell type-specific transcriptional programs in mouse prefrontal cortex during adolescence and addiction

Bhattacherjee et al.

SUPPLEMENTARY FIGURES

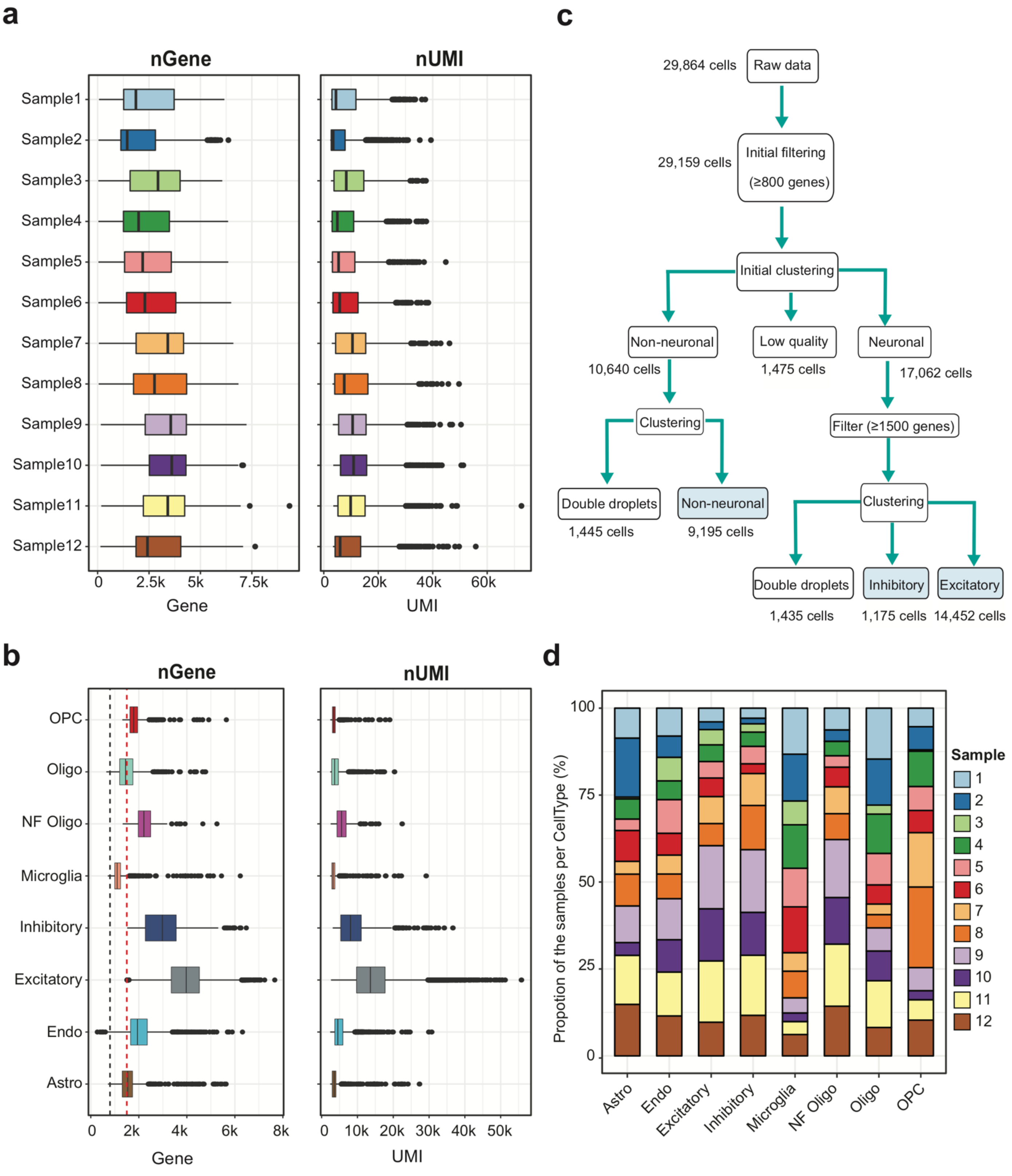

Supplementary Figure 1. PFC cell classification by scRNA-seq (related to Figure 1). a. Boxplots showing the distribution of the number of genes (left) detected in each of the 12 samples and corresponding UMI (right). **b.** Boxplot showing the distribution of the gene numbers (left) detected in each identified cell type across all samples and the corresponding UMI (right). The two dotted lines indicate 800 and 1,500 detected genes. **c.** Schematic showing the filtering steps and the number of cells obtained after filtering. **d.** Color-coded histogram showing percent of each of the 12 samples contributed to the detected cell clusters.

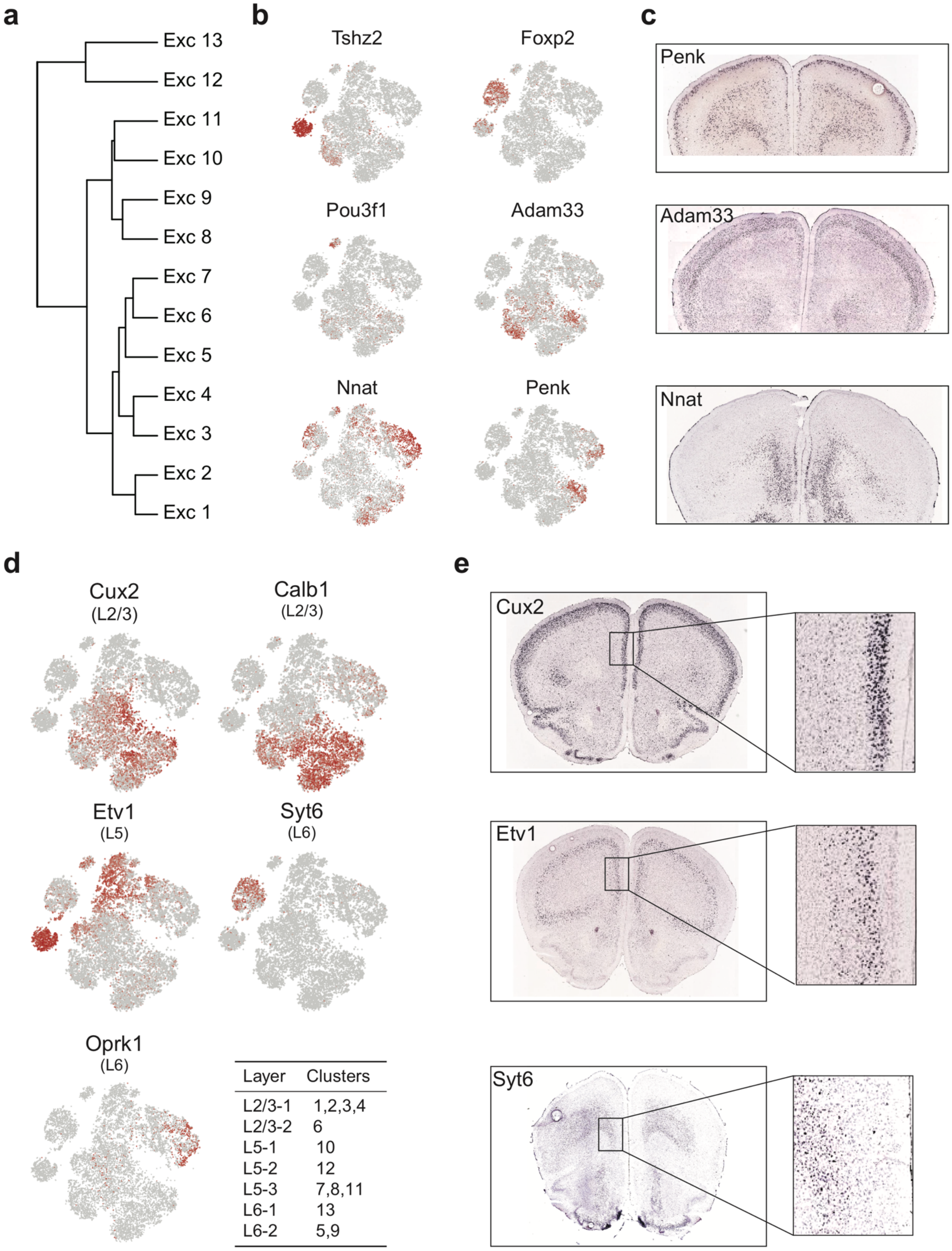

Layer	Clusters
L2/3-1	1,2,3,4
L2/3-2	6
L5-1	10
L5-2	12
L5-3	7,8,11
L6-1	13
L6-2	5,9

Supplementary Figure 2. Classification of PFC excitatory neurons and their assignment to cortical layers (related to Figure 2). a. Dendrogram showing hierarchical relationship between the excitatory neuron subtypes based on their transcriptome. **b.** t-SNE plots showing enrichment of selective markers in distinct excitatory neuron clusters. **c.** ISH images of coronal brain sections from mouse (P56) showing expression of the unique markers in distinct excitatory neuron populations (from Allen Brain Institute). **d.** Expression of specific markers of cortical layers projected on the excitatory t-SNE (*Cux2*- L2/3, *Calb1*- L2/3, *Etv1*- L5, *Sty6*- L6). Bottom right: Table summarizing the layer assignment of each cluster. **e.** Representative images showing distribution of the layer-specific markers in PFC (from the Allen Brain Atlas).

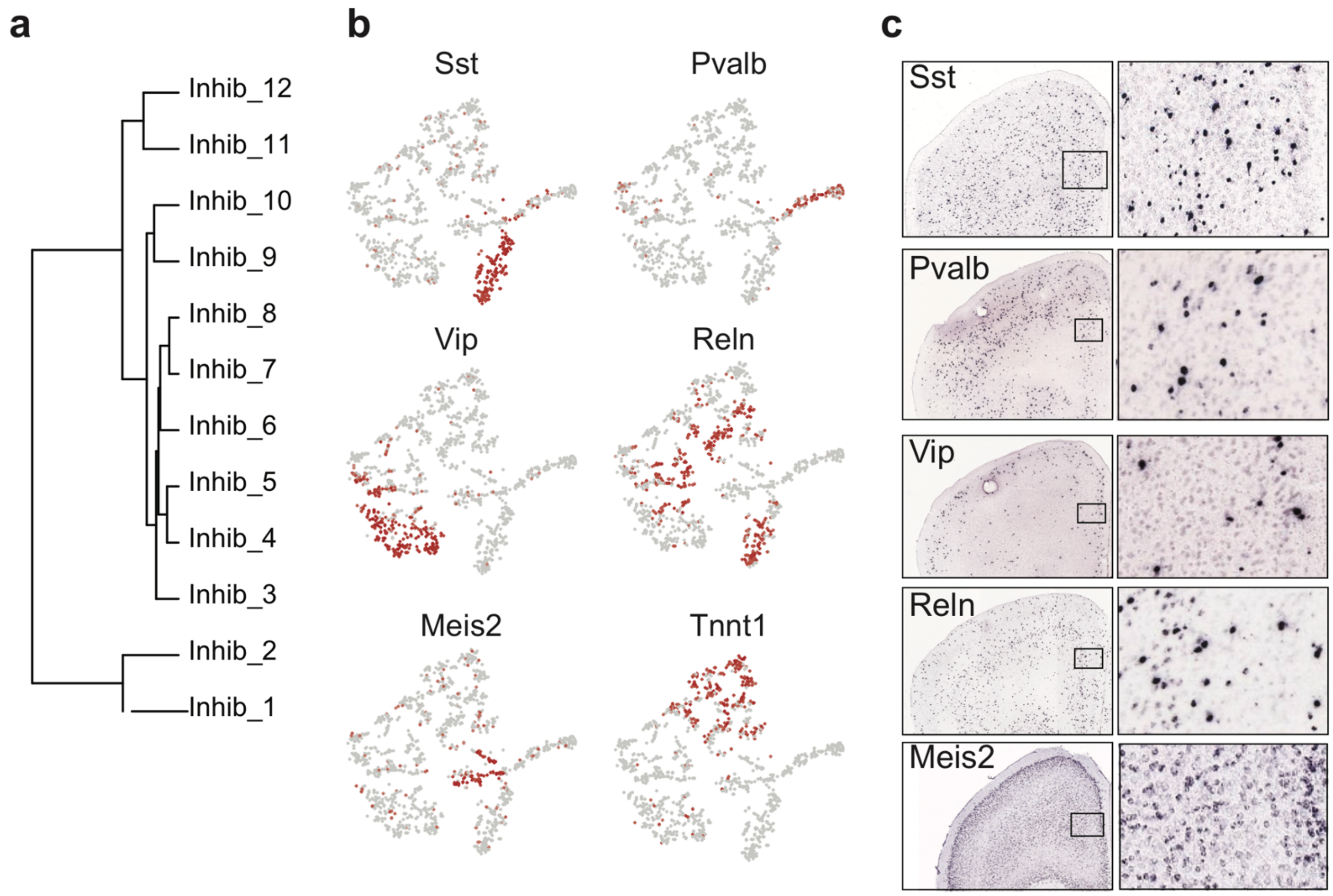

Supplementary Figure 3. Classification of PFC inhibitory neurons (related to Figure 2). a. Dendrogram showing hierarchical relationship between the inhibitory neuron subtypes. **b.** t-SNE plots showing enrichment of selective markers in distinct inhibitory neuron clusters. **c.** ISH images of coronal brain sections from mouse (P56) showing expression of the unique markers in distinct inhibitory neuron populations (from Allen Brain Institute).

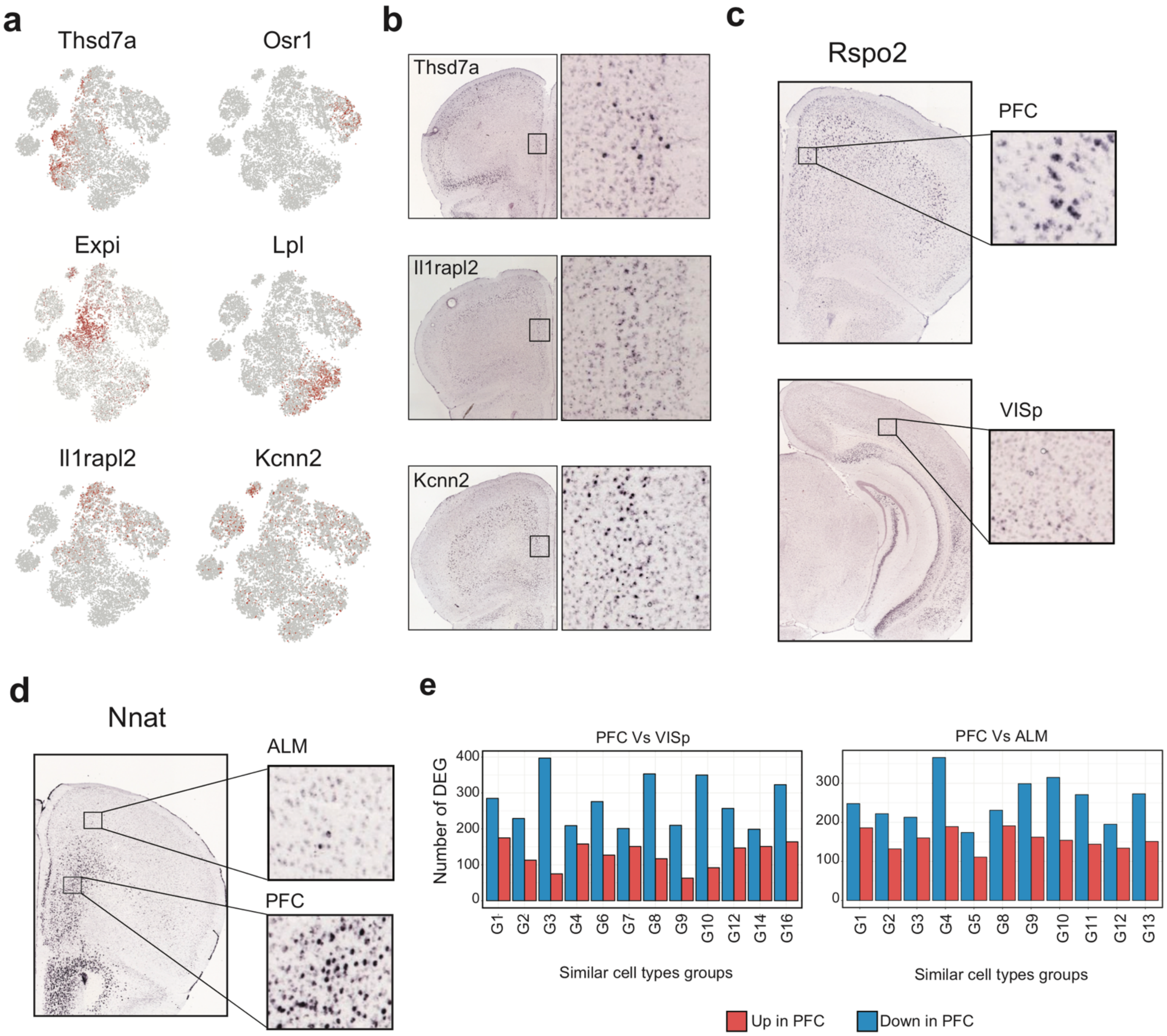

Supplementary Figure 4. Transcriptional properties of PFC excitatory neurons (Related to Figure 3). **a.** Distribution of representative cluster-specific markers on the t-SNE plot. **b.** ISH images showing tissue distribution of some of the cluster-specific markers in coronal brain sections (from Allen Brain Institute). **c.** ISH showing selective enrichment of *Rspo2* in PFC and depletion in VISp (from Allen Brain Institute). **d.** ISH images showing selective enrichment of *Nnat* in PFC and depletion in ALM (from Allen Brain Institute). **e.** Number of differentially expressed genes (FC>2, q-value<0.05, likelihood ratio test for zero-inflated data, Bonferroni corrected) among similar excitatory neuron subtypes detected in PFC when compared to VISp and ALM.

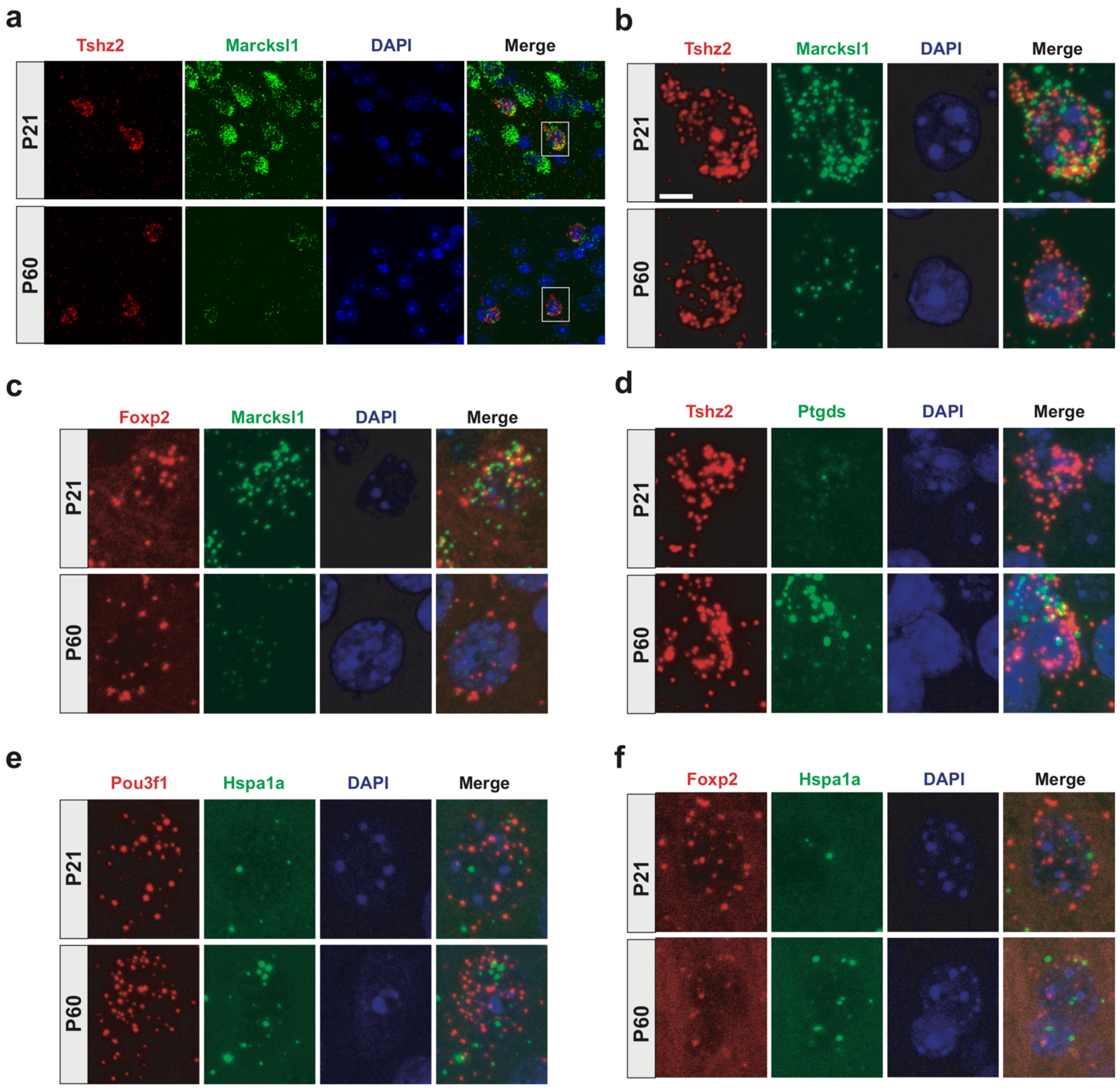

Supplementary Figure 5. Single molecule FISH validates scRNA-seq results (Related to Figure 4). a. Representative widefield images comparing FISH staining in P21 and P60 (candidate mRNA stained: *Marcksl1,* and *Tshz2* as a counter stain) **b.** Magnified view of single neurons from P21 and P60 in ‘a’ (white boxes in ‘a’ outline cells magnified) reveal signal from single RNA molecules validating reduced *Marcksl1* expression. **c.** Expression of *Marcksl1* is also selectively reduced in *Foxp2*$^{+}$ Ex-13 neurons. **d.** *Ptgds* expression is increased in *Tshz2*$^{+}$ Ex-12 neurons in P60 relative to P21. **e, f.** *Hspa1a* expression is increased in Ex-10 (marked by *Pou3f1*$^{+}$) and Ex-13 (marked by *Foxp2*$^{+}$) neurons in P60 relative to P21. (Scale bar in a=5μm)

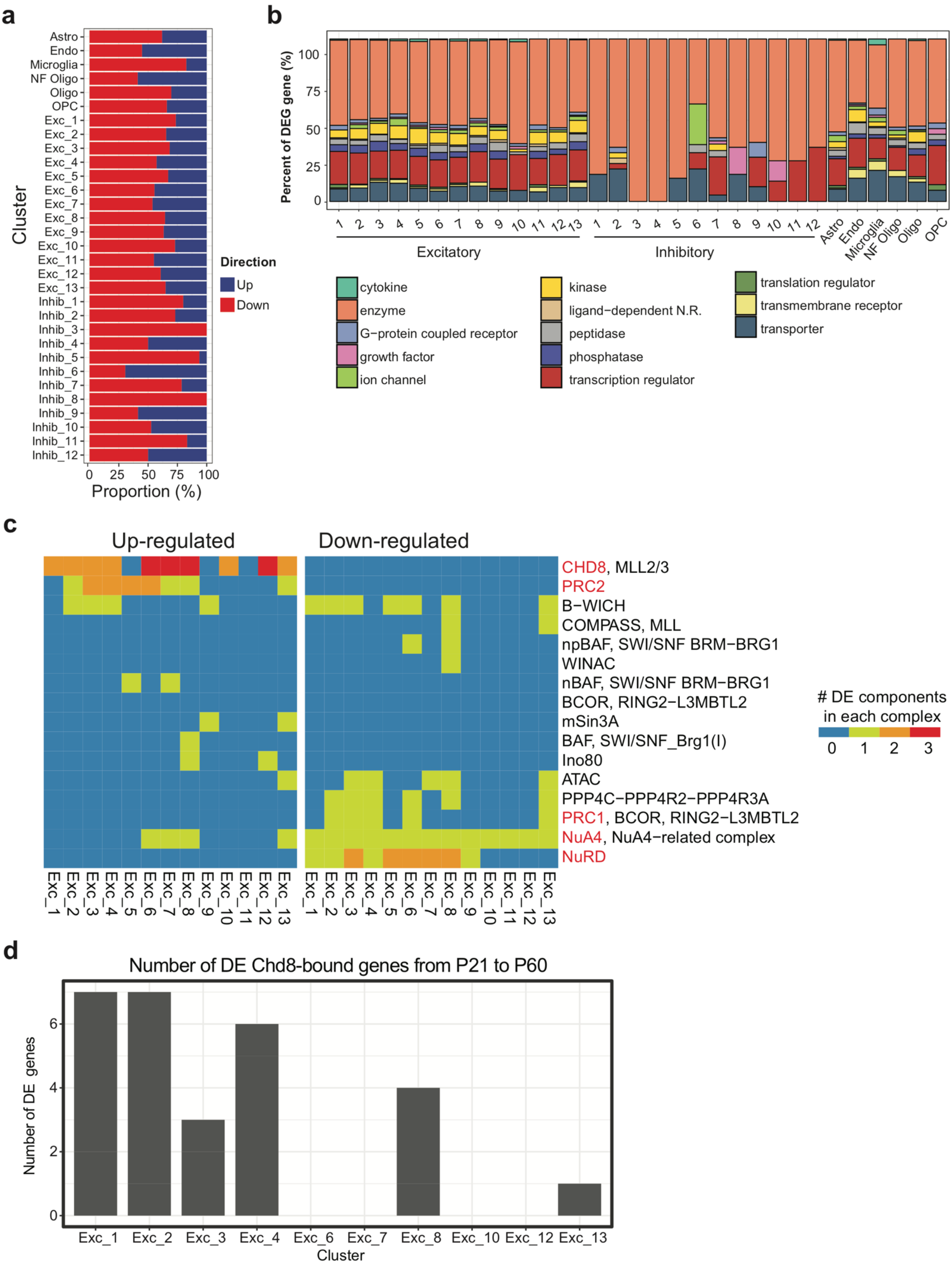

a
Cluster
Astro
Endo
Microglia
NF Oligo
Oligo
OPC
Exc_1
Exc_2
Exc_3
Exc_4
Exc_5
Exc_6
Exc_7
Exc_8
Exc_9
Exc_10
Exc_11
Exc_12
Exc_13
Inhib_1
Inhib_2
Inhib_3
Inhib_4
Inhib_5
Inhib_6
Inhib_7
Inhib_8
Inhib_9
Inhib_10
Inhib_11
Inhib_12
0 25 50 75 100
Proportion (%)
Direction
Up
Down
b
Percent of DEG gene (%)
0 25 50 75 100
1 2 3 4 5 6 7 8 9 10 11 12 13
Excitatory
1 2 3 4 5 6 7 8 9 10 11 12
Inhibitory
Astro Endo Microglia NF Oligo Oligo OPC
cytokine
enzyme
G−protein coupled receptor
growth factor
ion channel
kinase
ligand−dependent N.R.
peptidase
phosphatase
transcription regulator
translation regulator
transmembrane receptor
transporter
c
Up-regulated
Down-regulated
CHD8, MLL2/3
PRC2
B−WICH
COMPASS, MLL
npBAF, SWI/SNF BRM−BRG1
WINAC
nBAF, SWI/SNF BRM−BRG1
BCOR, RING2−L3MBTL2
mSin3A
BAF, SWI/SNF_Brg1(I)
Ino80
ATAC
PPP4C−PPP4R2−PPP4R3A
PRC1, BCOR, RING2−L3MBTL2
NuA4, NuA4−related complex
NuRD
Exc_1 Exc_2 Exc_3 Exc_4 Exc_5 Exc_6 Exc_7 Exc_8 Exc_9 Exc_10 Exc_11 Exc_12 Exc_13
Exc_1 Exc_2 Exc_3 Exc_4 Exc_5 Exc_6 Exc_7 Exc_8 Exc_9 Exc_10 Exc_11 Exc_12 Exc_13
DE components in each complex
0 1 2 3
d
Number of DE Chd8-bound genes from P21 to P60
Number of DE genes
0 2 4 6
Exc_1 Exc_2 Exc_3 Exc_4 Exc_6 Exc_7 Exc_8 Exc_10 Exc_12 Exc_13
Cluster

Supplementary Figure 6 Transcriptional dynamics in PFC cell clusters from P21 to P60 (related to Figure 4). a. Relative proportion of up- and down-regulated genes in each PFC cell type and cell clusters between P21 and p60 (FC>1.5, q-value<0.05, negative binomial generalized linear model, Bonferroni corrected). **b.** Proportions of the different molecular classes dynamically regulated between P21 and P60. **c.** Heatmap showing the number of different epigenetic modifier complexes whose members are up- or down-regulated between P21 and P60 PFC excitatory neuron clusters. **d.** The number of *Chd8* target genes whose expression changed between P21 and P60 in different excitatory clusters.

a

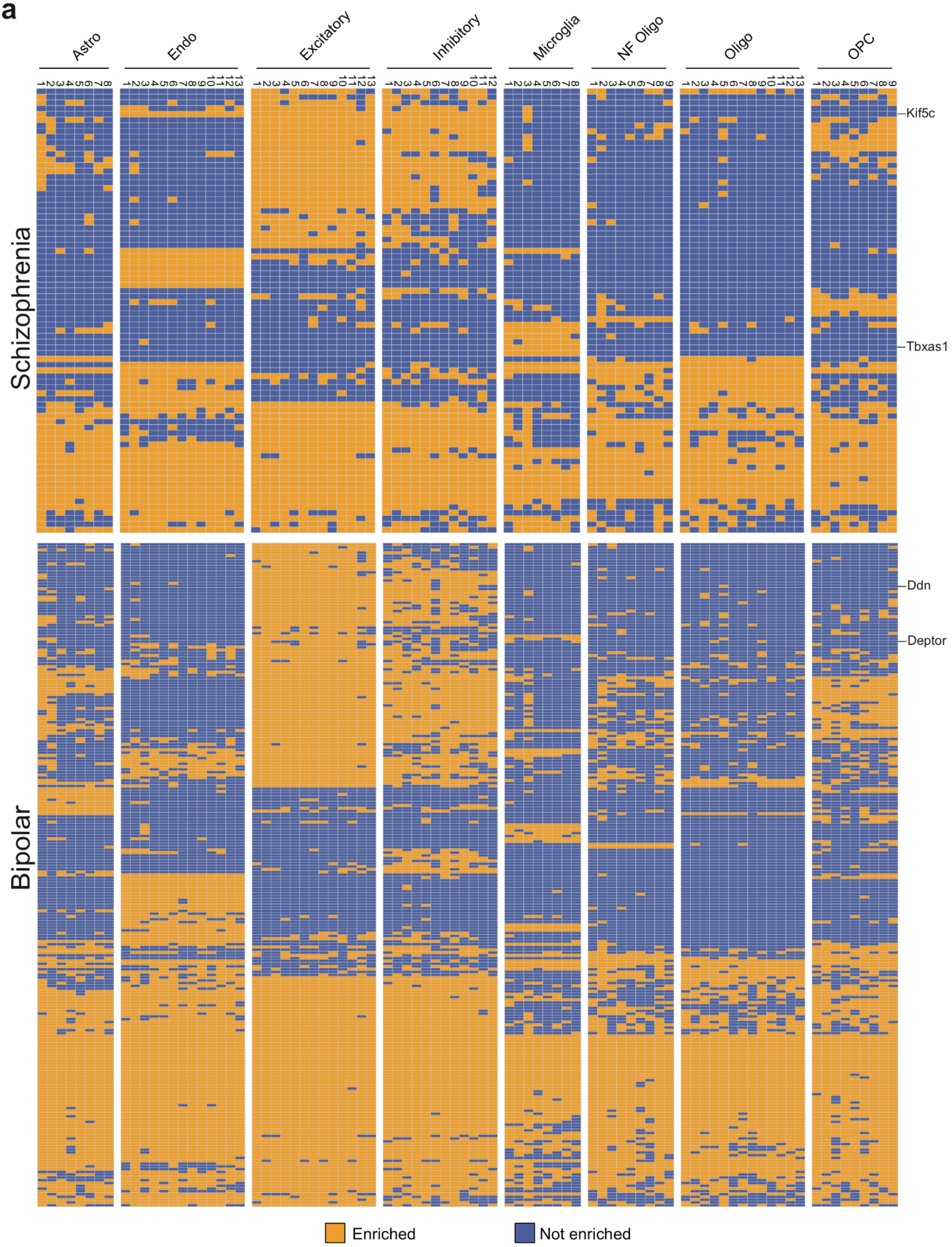

Astro
Endo
Excitatory
Inhibitory
Microglia
NF Oligo
Oligo
OPC
Schizophrenia
Bipolar
Kif5c
Tbxas1
Ddn
Deptor
Enriched
Not enriched

Supplementary Figure 7 Expression of schizophrenia and bipolar disorders GWAS candidate genes in PFC cell clusters (related to Figure 5). Heat map showing the expression of GWAS candidate genes relevant to schizophrenia and bipolar disorders in each PFC cell clusters. The gene list can be found in Supplementary Data 6.

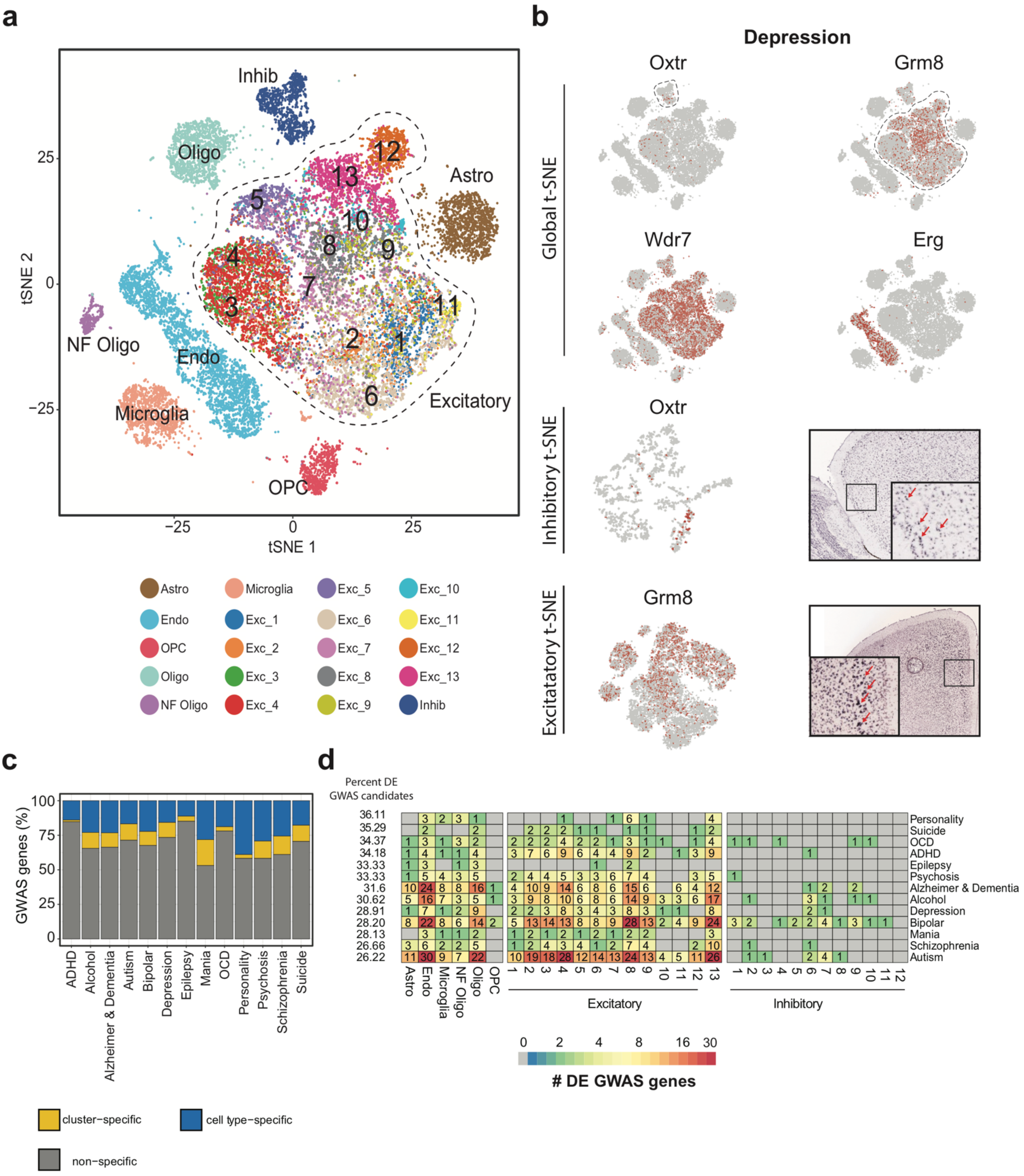
a
b
Depression
Oxtr
Grm8
Global t-SNE
Wdr7
Erg
Inhibitory t-SNE
Excitatatory t-SNE
Inhib
Oligo
Astro
Excitatory
NF Oligo
Endo
Microglia
OPC
tSNE 1
tSNE 2
Astro
Endo
OPC
Oligo
NF Oligo
Microglia
Exc_1
Exc_2
Exc_3
Exc_4
Exc_5
Exc_6
Exc_7
Exc_8
Exc_9
Exc_10
Exc_11
Exc_12
Exc_13
Inhib
c
GWAS genes (%)
ADHD
Alcohol
Alzheimer & Dementia
Autism
Bipolar
Depression
Epilepsy
Mania
OCD
Personality
Psychosis
Schizophrenia
Suicide
cluster−specific
cell type−specific
non−specific
d
Percent DE GWAS candidates
Personality
Suicide
OCD
ADHD
Epilepsy
Psychosis
Alzheimer & Dementia
Alcohol
Depression
Bipolar
Mania
Schizophrenia
Autism
Astro
Endo
Microglia
NF Oligo
Oligo
OPC
Excitatory
Inhibitory
DE GWAS genes

Supplementary Figure 8 Cluster-specific expression of GWAS candidates and their dynamical regulation during adolescence (related to Figure 5). a. Identification of different cell subtypes (particularly the excitatory) within the global t-SNE of all PFC cells **b.** t-SNE-plot showing examples of some cell type and subtype specific expression of GWAS candidate genes in depression. Top-panel shows the gene expression enrichment of four representative candidates on the global t-SNE plot. The bottom panels show zoomed t-SNE of excitatory and inhibitory clusters, respectively, and Allen Brain ISH images showing the specific gene expression. **c.** Percentage of selective, broadly selective and non-selective GWAS candidate genes for each of the 12 disorders analyzed. **d.** Heat map showing the number of differentially expressed GWAS candidate genes relevant to the 12 PFC related diseases in the PFC excitatory, inhibitory clusters and non-neuronal cell types between P21 and P60 mice.

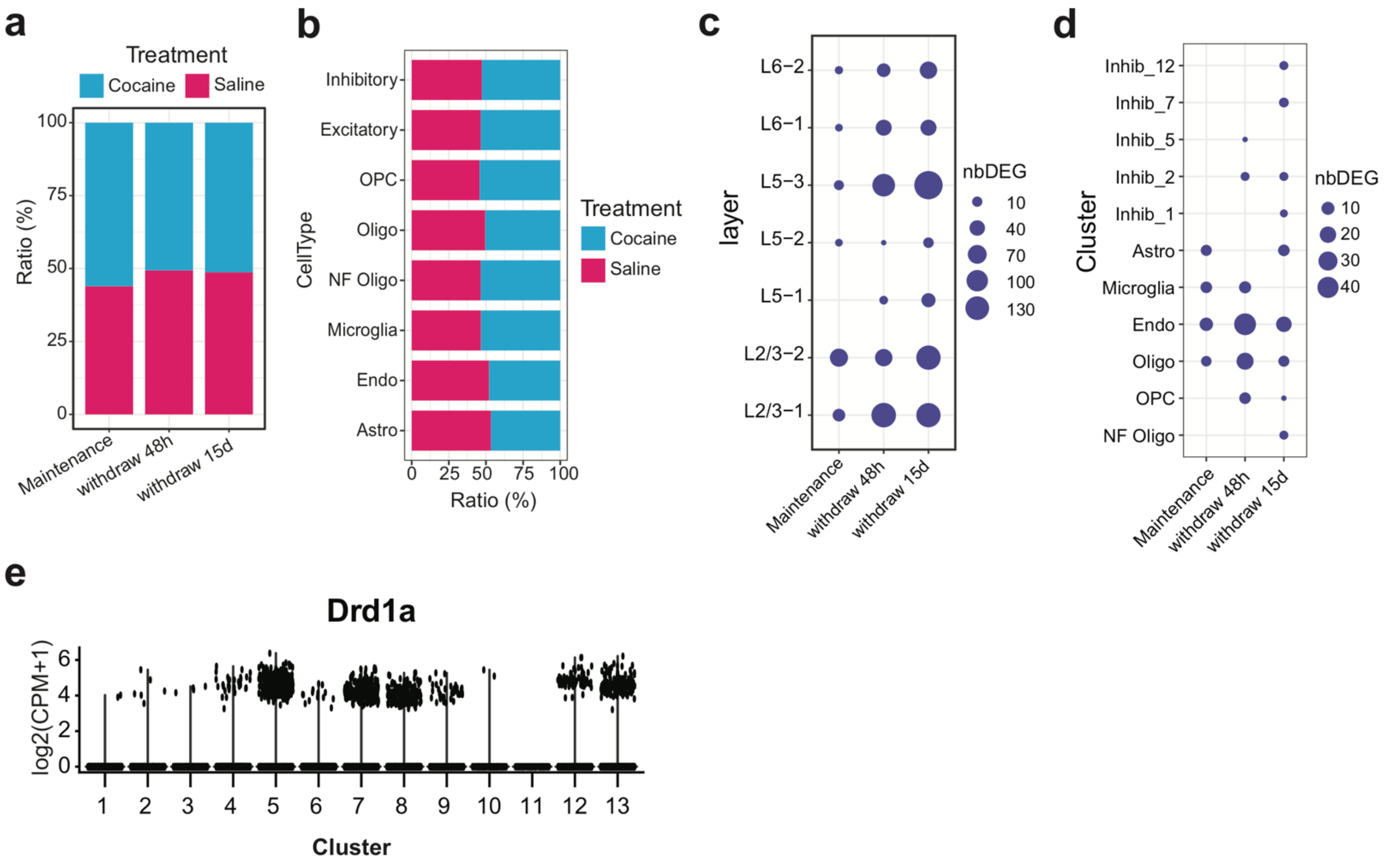

Supplementary Figure S9 Behavioral and transcriptional outcomes in chronic cocaine IVSA (Related to Figure 6). a. Percentage of cells contributed by the saline and cocaine groups to data at each time point of sample collection. **b.** Percentage of cells contributed by saline and cocaine groups towards each basic cell types. **c.** Number of differentially expressed genes aggregated by excitatory layers of PFC in each time point of sample collection. **d.** Number of differentially expressed genes in the major inhibitory neuron subtype and non-neuronal cell types between saline and cocaine groups at the three time points of sample collection. **e.** Expression of *Drd1* in excitatory neuron subtypes. The differentially expressed genes in c and d where calculated using FC>1.5 and SC2P model phase2 FDR <0.05, empirical Bayes statistic, limma package.

a

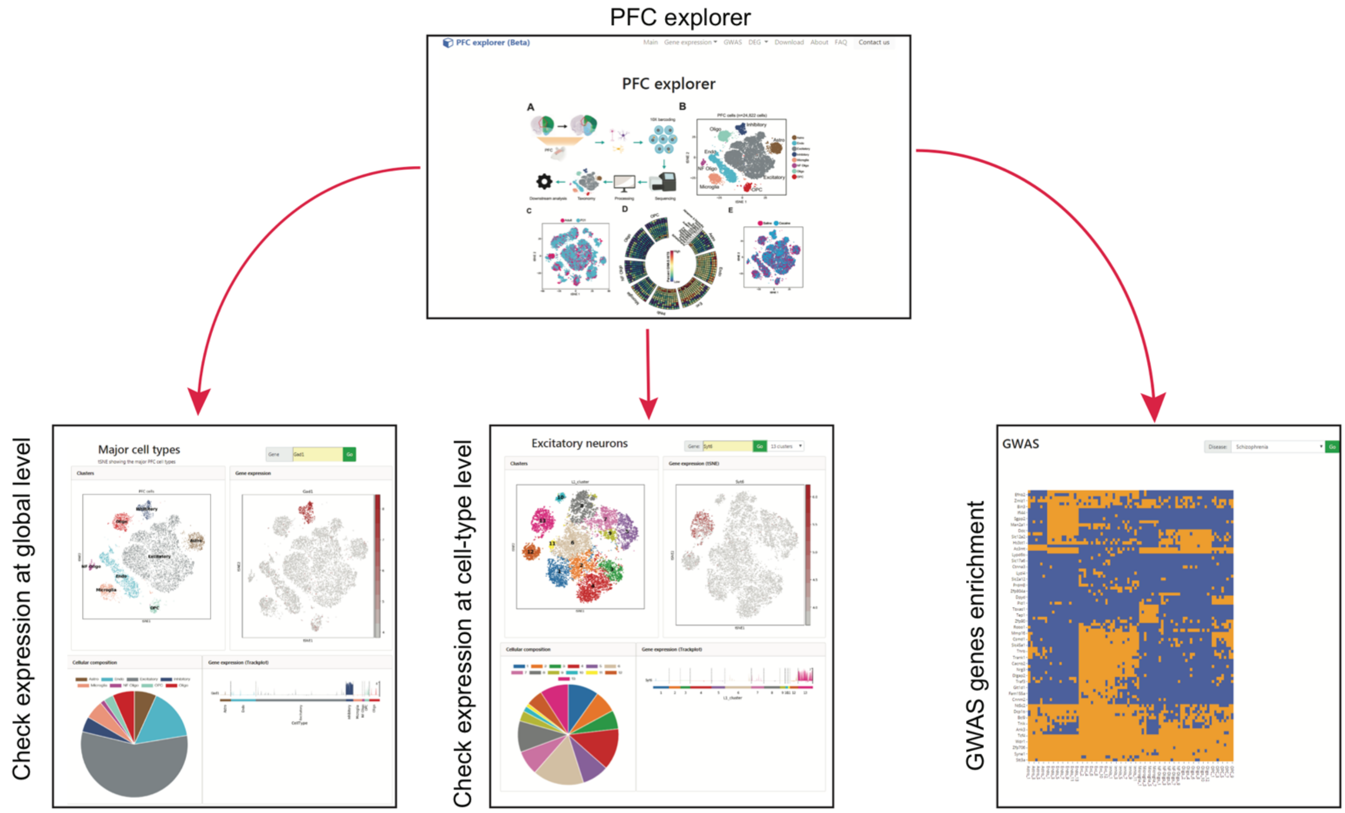

Supplementary Figure 10. Web-portal for the interactive exploration of the PFC data. An interactive web-portal that enables the exploration of the analyzed data is developed. Users can check the expression of genes at the global level (bottom left), cell-type level (bottom middle) and can also explore the enrichment of 12 sets of disease-associated genes in the different PFC cell types. Computer and mouse in top panel were created using BioRender.com.

Supplementary Data:

Supplementary Data 1. Summary of the mapping statistics of the single-cell libraries

Supplementary Data 2. The number of cells and sub-clusters for each cell type

Supplementary Data 3. List of genes enriched in PFC compared to VISp and ALM and list of the original cluster names corresponding to the abbreviations in Fig.3C.

Supplementary Data 4. List of differentially expressed genes in each cell type and cell subcluster between P21 and P60

Supplementary Data 5. List of differentially expressed epigenetic factors in each cell type and cell subcluster between P21 and P60

Supplementary Data 6. Expression status of PFC-relevant disease GWAS candidate genes

Supplementary Data 7. List of differentially expressed genes in cocaine IVSA
