# Supplementary material for: Cell type-specific transcriptional programs in mouse prefrontal cortex during adolescence and addiction
Source: Nat Commun. 2019 Sep 13;10:4169. doi: 10.1038/s41467-019-12054-3 (PMC6744514; doi:10.1038/s41467-019-12054-3)
Supplement: Supplementary file 2 — Description of Additional Supplementary Files [file 41467_2019_12054_MOESM2_ESM.docx]

**Description of Additional Supplementary Files**

**Supplementary Data 1:** Mapping statistics of the different single-cell libraries

**Supplementary Data 2:** Number of cells detected by cell-type

**Supplementary Data 3-1:** Correspondance between the original VISp and ALM cluster names and abreviated names in Fig.3C

**Supplementary Data 4:** Differential gene expression results for different clusters between P21 and P60

**Supplementary Data 5-2:** list of the differential Chd8 targets between P21 and P60

**Supplementary Data 6:** Table summarizing the enrichment of the different disease-associated GWAS candidate genes

**Supplementary Data 7:** Differential expression results after cocain IVSA in different phases
